# Ethical dimensions and societal implications: ensuring the social responsibility of CRISPR technology

**DOI:** 10.3389/fgeed.2025.1593172

**Published:** 2025-08-20

**Authors:** Irfan Biswas

**Affiliations:** ^1^ University of Massachusetts School of Medicine, Worcester, MA, United States; ^2^ Shrewsbury Sr. High School, Shrewsbury, MA, United States; ^3^ University of Rhode Island, Kingston, RI, United States

**Keywords:** CRISPR- Cas 9, gene editing, bioethics, genetic inequality, eugenics, global health equity, genome regulation, ethical implications of biotechnology

## Abstract

CRISPR-Cas9 is a breakthrough genome-editing platform that can cut chosen DNA sequences with unprecedented speed, accuracy, and affordability. By reprogramming a single guide RNA, researchers now alter gene function, correct pathogenic variants, or introduce novel traits. Earlier tools such as zinc-finger nucleases and TALENs performed similar tasks but were significantly more complex and costly. Yet CRISPR’s very power raises urgent ethical concerns: Who controls its use, and how can society prevent germ-line enhancement, eugenic selection, or unequal access that favors wealthy nations and patients? A well-publicized case of embryo editing already showed how premature, unregulated experiments can erode public trust. This perspective therefore frames CRISPR’s scientific promise alongside its social responsibilities, arguing that proactive, globally coordinated governance is essential to unlock benefits while preventing new forms of genetic inequality.

## The social responsibility of CRISPR

**FIGURE 1:**
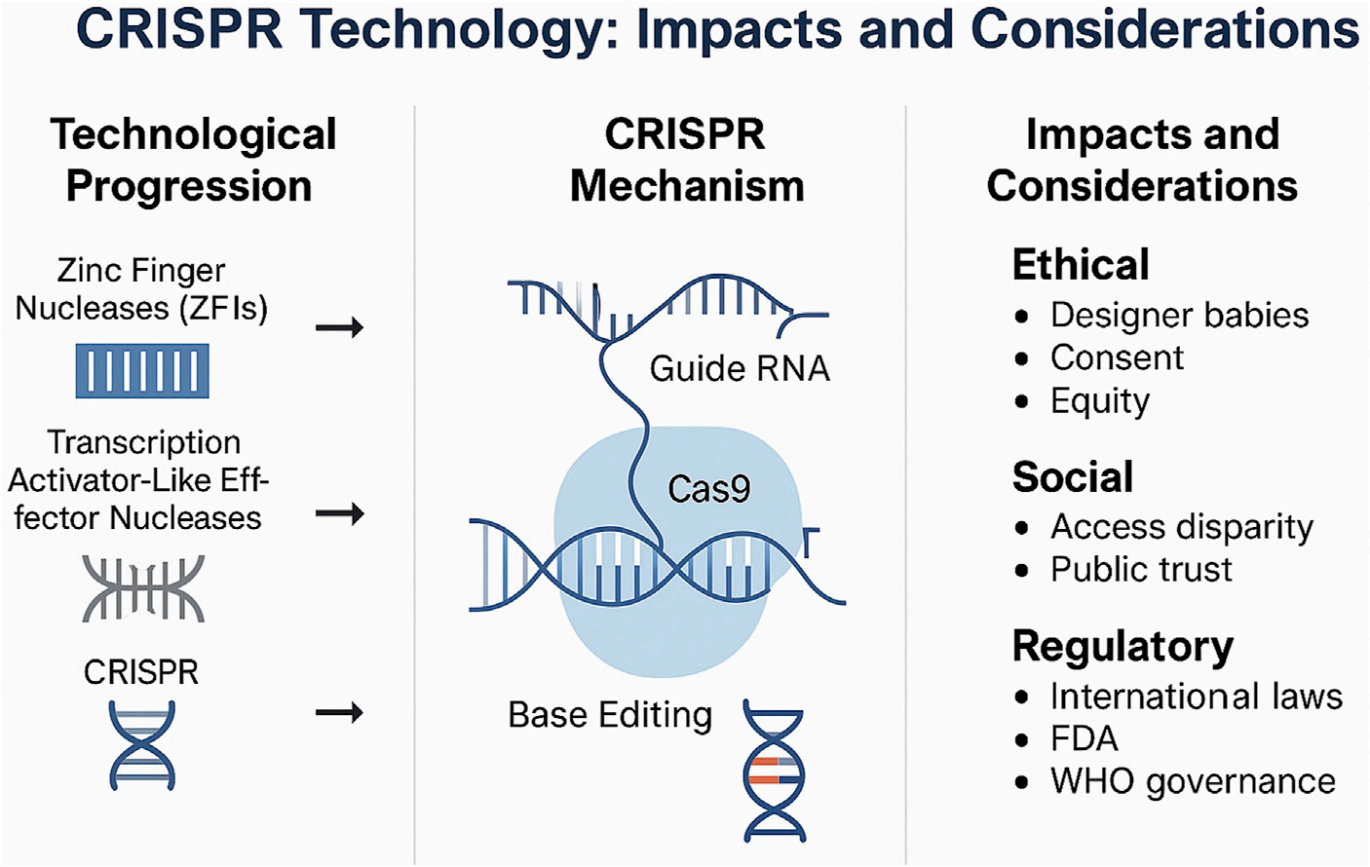
Overview of CRISPR technology, its progression, mechanism, and key ethical, social, and regulatory considerations.

CRISPR gene editing, or clustered regularly interspaced short palindromic repeats, is a revolutionary scientific advancement which has incredible potential. This modern technology allows scientists and researchers to single handedly cut precise DNA sequences, after determining the locations of each sequence, offering the potential to change characteristics and the makeup of proteins. Prior to the development of CRISPR, gene editing technologies such as zinc finger nucleases (ZFNs) and transcription activator-like effector nucleases (TALENs) were employed to modify DNA. However, these earlier methods were often complex, time-consuming, and costly. CRISPR-Cas9 revolutionized the field by offering a more efficient, programmable, and accessible approach to genome editing (Figure 1).

The possibilities are endless as CRISPR can be applied to medicine, agriculture, and even environmental science to combat climate change. However, the efficiency and cost-effectiveness of the technology still raises significant ethical and social responsibilities. With the ability to alter human characteristics, and bar certain populations due to the cost of the technology raises further questions about the equity and access of the machine. Hence, while CRISPR has potential for playing a key role in curing genetic diseases, increasing crop yields, and combating climate change, the misuse of the technology may deepen social divide, create monopolies, and decrease genetic diversity. For these reasons, it is crucial for governments and corporations to come together to use CRISPR technology responsibly and equitably to benefit everyone.

This technology has enabled scientists to precisely edit genes and certain characteristics throughout multiple fields and has propelled the field of biotechnology. In the medical field, CRISPR is able to treat generation-long genetic disorders that were once thought to be incurable, providing hope for patients suffering from a multitude of genetic diseases. Specifically, in *CRISPR: Can We Control It?,* Doudna and her team discuss the application of CRISPR in genetic diseases like sickle cell anemia and cystic fibrosis by directly locating and cutting out the mutations that caused these conditions (Doudna et al., 2021). Beyond inducing double-stranded DNA breaks, CRISPR-based technologies have expanded to include advanced techniques such as base editing and prime editing. These tools allow for precise conversion of individual DNA bases without creating breaks in the DNA strand. For instance, base editors can convert C⋅G base pairs to T⋅A, offering a powerful strategy for correcting point mutations linked to genetic disorders. These innovations greatly broaden the therapeutic and research applications of CRISPR. Additionally, the technology is now being applied to cancer treatment by enhancing immune cells and their ability to locate and destroy cancerous growth. Evidently, the applications of CRISPR can save hundreds of thousands, if not millions, of lives.

Nonetheless, the applications of CRISPR extend beyond genetic diseases and cancerous development. Additional conditions such as heart disease and diabetes can be tackled through the use of gene editing, showcasing the expansive applications of CRISPR technology in medicine. Furthermore, CRISPR-mediated gene editing enables the precise targeting of the underlying genetic causes of disease, thereby facilitating patient-specific interventions. This enhanced specificity not only improves therapeutic efficacy by enabling accurate identification of pathogenic gene sequences but also reduces off-target effects, ultimately minimizing adverse side effects.

However, CRISPR has applications beyond just the medical field. As climate change becomes an increasingly more prevalent threat to the globe, it has also taken tolls on food. With rising temperatures and extreme weather, there has been a decline in crop yields and a shift in the development of staple foods. Huang et al. discusses an example with CRISPR and its ability to modify Penicillium, a species of fungi. Often regarded as a staple food and key agricultural resource, it was crucial to develop resistance to the extreme weather in order to enhance crop resilience and productivity. For example, gene-edited crops are enabled with the ability to withstand drought and pests, ensuring stable food supplies throughout the season, even with harsh weather conditions (Huang et al., 2024). Through these advancements, the role of CRISPR has become increasingly prevalent in solving global challenges. Still, this technology also raises important questions about who and how to control this revolutionary technology.

Hence, while the potential of CRISPR may be undeniable, the applications of CRISPR can still be abused and cause major risk. As Doudna warns, the same technology for curing generation-long genetic diseases can also be used to select “superior” human traits, and create monopoly organizations. The potential and concerns regarding this technology highlight the need for ethical considerations on its ability to aid society as a whole, without sacrificing social equity or ethical integrity.

A major ethical concern surrounding CRISPR and its ability to integrate into society is the potential for it to exacerbate social inequality. In *CRISPR People: The Science and Ethics of Editing Humans*, Henry Greeley warns of the dystopian society that gene editing can create, where a genetic “underclass” may be created due to wealthier individuals being able to access and create a population with “superior traits” and any other enhancements that may be barred to others due to the significant cost of the technology. For example, affluent families may use CRISPR to engineer their children with specific traits relating to enhanced intelligence or athletic capability. Consequently, this can lead to an entire society, possibly a world, where genetic privilege bars others from social mobility and creates generational disparities (Greely, 2021). In fact, one of the most pressing ethical concerns surrounding CRISPR is its potential to revive eugenic ideologies through the selection or enhancement of genetic traits. While therapeutic gene editing offers immense potential, the use of CRISPR for non-medical enhancements—such as intelligence, physical traits, or predisposition to talent—raises the risk of deepening social inequalities and creating a genetic underclass. The 2018 case of the “CRISPR babies” in China, where a scientist edited the genes of twin embryos to confer resistance to HIV, drew intense international condemnation due to the ethical violations and lack of regulatory oversight. This incident serves as a cautionary tale of how premature and unregulated CRISPR experimentation can pose significant societal risks, emphasizing the need for global ethical frameworks to prevent misuse and ensure equity (Regalado, 2018).

Greely’s argument should be considered for the worst case scenario, if CRISPR should take over and truly become misused by the government and our society. However, with proper regulations and oversight, the commercialization of CRISPR should not mirror a dystopian society of genetic classes, instead it should be used as a tool for accessible and equitable healthcare and education. Therefore, it is crucial to develop international and corporate frameworks to ensure CRISPR continues to address pressing global challenges, and should not be used as a tool that might become a luxury product leaving marginalized communities behind. Through international frameworks of equitable access to the technology, CRISPR will be able to continue addressing genetic diseases, and combat food scarcity without increasing social divides.

Another ethical concern regarding the misuse of CRISPR is the ability to choose traits that may be a result of social stigmas. In *Time* magazine, Kozubek argues the desire to create “designer babies”, and the risks to the overall human population as a result of this. For instance, cultural preferences for certain physical features or abilities may lead to enhanced traits and the reduction of other traits that may be undesirable in humans. As a result, these genetic modifications for “better” features will result in a loss in genetic variability and the capability for humans to adapt to various environments and diseases (Kozubek, 2017). This loss of diversity can have greater consequences than creating a more genetically enhanced population. From an evolutionary standpoint, the less genetic diversity means humans will have greater efforts towards adapting towards various changes in the environment. Whether it be a virus, such as the COVID-19 pandemic, or changes in weather, the loss of genetic diversity can create more vulnerability to extinction by directly hindering evolutionary potential and genetic diversity. Additionally, the risk of creating similar characteristics and genes throughout society can increase the risk of inbreeding, and can potentially make certain diseases more prevalent and susceptible to greater numbers of people, essentially causing lower adaptability to various environmental conditions.

Additionally, the ability to determine biological traits in humans adds a whole layer of ethical considerations by itself. Decisions about genetic modifications can be influenced by cultural, political, or even economic purposes that can cause further social divide and directly put marginalized communities behind. Hence, it is important to ensure that the development of CRISPR and its policies are used responsibly and inclusively.

A key responsibility for ensuring the ethical and equitable use of CRISPR also depends on the stakeholders, corporations, and governments that have control of the technology. Scientists also have a key role in determining the future of this technology. A key contributor towards the development of the technology, Doudna and her colleagues emphasize the importance of transparency and public engagement in the development of CRISPR. For example, researchers can work alongside sociologists and policymakers to establish specific and equitable guidelines that prioritize social responsibility. By creating open dialogue between scientists and policymakers who understand societal needs, CRISPR can be created in ways that align with societal values and needs.

Corporations are another key player in the commercialization of CRISPR, and must also be held socially accountable. Greely argues that private companies should not have unchecked control over CRISPR technology, instead governments must implement policies in order to regulate the use of CRISPR and prevent the misuse of the technology. For example, regulatory frameworks can allow corporations to require making CRISPR treatments for genetic diseases and agricultural products at affordable prices in order to allow marginalized communities and potentially low-income nations to have access to this revolutionary technology. Additionally, international cooperation would allow for addressing global challenges CRISPR may be able to address. Additionally, it is crucial to include lower-income countries and make inclusive decisions that involve both developed and developing nations in order to create equitable access to the technology globally. For instance, global peace organizations such as the United Nations can coordinate efforts and establish universal ethical standards for CRISPR use. Through international and corporate frameworks, CRISPR can balance the benefits and the risks related to the technology, and create equitable access to the technology (Rajgarhia et al., 2024).

Despite the various benefits of the technology, many critics argue that the risks may outweigh the benefits. For example, Kozubek explains his concerns about the long-term consequences of gene editing and its potential to disrupt natural evolutionary processes and create unforeseen biological risks we may not be aware of. Although these concerns may be significant, alone, they do not justify abandoning a technology with the capability of addressing a multitude of global issues. Instead, these concerns highlight the need and importance of strict protocol and regulatory frameworks to mitigate the risks of unforeseen biological risks, or a decrease in overall genetic diversity. Governments have the capacity to play a pivotal role in the regulation of CRISPR-based technologies by procuring such products and subjecting them to stringent preclinical and clinical evaluation. Through comprehensive testing and ongoing surveillance, regulatory bodies can ensure the safety, efficacy, and ethical compliance of CRISPR applications prior to their widespread adoption. As a result, these steps can help mitigate any unintended consequences of the technology while also preserving the potential of CRISPR to improve human wellbeing.

Another important consideration is that CRISPR may be inevitable, as it is currently only accessible to wealthy nations and individuals, pricing at a significant $2.2 million per patient. Rajgarhia highlights the potential for both commercialization and equitable distribution of CRISPR technologies through strategic international investment, particularly in the context of developing countries. For instance, global institutions such as the United Nations could spearhead initiatives aimed at advancing research into cost-effective CRISPR-based therapies and agricultural innovations, thereby promoting broader accessibility and global health equity. These efforts can help overcome the significant costs of the technology and create an initiative for equitable access to CRISPR globally. Additionally, the urgency of establishing transparent, inclusive, and enforceable global policies for CRISPR technology is becoming increasingly apparent. A recent Nature editorial underscores the growing divide in how different countries are approaching gene editing, particularly in human embryos. It stresses the need for international cooperation to prevent ethical gray zones, regulatory loopholes, and misuse of CRISPR in ways that may deepen global health inequities (Nature, 2025). These concerns directly align with this review’s focus on ensuring that CRISPR advances are guided not only by scientific potential, but also by a shared ethical framework.

CRISPR gene editing is an important tool which has made groundbreaking advancements in science, agriculture, and various other fields. With the ability to cure genetic diseases and address various other global challenges such as climate change, ethical responsibilities and considerations also play an important role. The ability for CRISPR to alter entire human characteristics and agriculture can on one hand immensely benefit society. On the other hand, the risks include deepening social divides and decreasing overall human genetic diversity. To ensure CRISPR is used for the overall common good, it is essential to incorporate ethical guidelines that prioritize equity, transparency, and accountability globally. Additionally, the cooperation between corporations and governments is just as important as they must work together to discuss and address the ethical and accessibility challenges associated with CRISPR internationally. Although there may be some risks involved with the development and distribution of CRISPR technologies, it is crucial to continue progressing the development of CRISPR to ensure it is used ethically, equitably, and for the greater good.

## References

[B1] Doudna J. A. Charpentier E. Jinek M. (2021). CRISPR: can we control it? Big think.

[B2] Greely H. T. (2021). CRISPR people: the science and Ethics of editing humans. MIT Press.

[B3] Huang Y. Li X. Wang Z. Qi G. Hao L. Xin T. (2024). Analysis of whole-genome for identification of seven Penicillium species with significant economic value. Int. J. Mol. Sci. 25 (15), 8172. 10.3390/ijms25158172 39125741 PMC11312406

[B4] Kozubek J. (2017). How CRISPR and gene editing could ruin human evolution. New York, United States: Time Inc. Available online at: https://time.com/4626571/crispr-gene-modification-evolution/.

[B5] Rajgarhia A. Sharma R. Patel V. Sachdev S. Sravan T. Singh N. (2024). National innovation system for CRISPR genome editing research in India: an empirical analysis. Curr. Sci. 127 (6), 674–683. 10.18520/cs/v127/i6/674-683

[B6] Regalado A. (2018). Exclusive: chinese scientists are creating CRISPR babies. MIT Technology Review. Available online at: https://www.technologyreview.com/2018/11/25/138962/exclusive-chinese-scientists-are-creating-crispr-babies/.

[B7] Nature (2025). We need to talk about human genome editing Nature. 625, 7. 10.1038/d41586-025-00015-4 39780015

